# β Oscillation during Slow Wave Sleep and Rapid Eye Movement Sleep in the Electroencephalogram of a Transgenic Mouse Model of Huntington’s Disease

**DOI:** 10.1371/journal.pone.0079509

**Published:** 2013-11-14

**Authors:** Yannick Jeantet, Sebastien Cayzac, Yoon H. Cho

**Affiliations:** 1 University of Bordeaux, Talence, France; 2 Institut de Neurosciences Cognitives et Integratives de l’Aquitaine, CNRS UMR 5287, Talence, France; Hôpital du Sacré-Coeur de Montréal, Canada

## Abstract

**Study objectives:**

To search for early abnormalities in electroencephalogram (EEG) during sleep which may precede motor symptoms in a transgenic mouse model of hereditary neurodegenerative Huntington’s disease (HD).

**Design:**

In the R6/1 transgenic mouse model of HD, rhythmic brain activity in EEG recordings was monitored longitudinally and across vigilance states through the onset and progression of disease.

**Measurements and results:**

Mice with chronic electrode implants were recorded monthly over wake-sleep cycles (4 hours), beginning at 9–11 weeks (presymptomatic period) through 6–7 months (symptomatic period). Recording data revealed a unique β rhythm (20–35 Hz), present only in R6/1 transgenic mice, which evolves in close parallel with the disease. In addition, there was an unusual relationship between this β oscillation and vigilance states: while nearly absent during the active waking state, the β oscillation appeared with drowsiness and during slow wave sleep (SWS) and, interestingly, strengthened rather than dissipating when the brain returned to an activated state during rapid eye movement (REM) sleep.

**Conclusions:**

In addition to providing a new in vivo biomarker and insight into Huntington's disease pathophysiology, this serendipitous observation opens a window onto the rarely explored neurophysiology of the cortico-basal ganglia circuit during SWS and REM sleep.

## Introduction

Huntington’s disease (HD) is a fatal hereditary neurodegenerative disease associated with chorea and motor disturbances. The disease is caused by mutation of a gene encoding for huntingtin protein [1]. Although expression of the huntingtin protein is ubiquitous, the striatum and cortex in particular are affected early in the disease [2]. Moreover, the first symptoms of this progressive disease may not be movement disturbances, but deficits in learning and memory [3], [4]. In addition, most HD patients and transgenic mice exhibit sleep and circadian cycle disturbances [5], [6], [7], [8], [9].

Our previous studies with the R6/1 transgenic mouse model of HD [10] demonstrated enhanced β oscillation and altered striatal activity associated with impaired procedural learning ability [11], and disrupted segregation between active and resting brain states during sleep [12]. To detect early changes in sleep electroencephalogram (EEG) that could be used as a biomarker of the HD pathology, we longitudinally monitored sleep EEG in R6/1 mice throughout disease onset and progression, along with motor symptoms.

## Materials and Methods

### Ethics Statement

All experimental procedures were approved by the local Institutional Animal Care and Use Committee (Comité d’Ethique pour l’Expérimentation Animale Bordeaux), and were in accordance with the European Communities Council Directive of 24 November 1986 (86/609/EEC).

### Subjects

Subjects were male R6/1 transgenic mice (n = 10) and age-matched wild-type (WT) littermates (n = 8) aged 8–9 weeks at the beginning of the experiment (i.e., the time of surgery). Mice were obtained by crossbreeding male R6/1 mice (B6.Cg-Tg (HDexon1) 61Gpb/J, Stock number: 006471, Jackson Laboratory, Main Harbor, NY, USA) and female C57BL6/J mice (IFFA/Credo, Lyon, France). The R6/1 line expresses exon 1 of the human HD gene with an expanded number of CAG trinucleotide repeats (approximately 126 repetitions). Genotypes were tested by PCR of tail biopsy specimens. All animals were housed individually in polycarbonate standard cages (33×15×14 cm; Tecniplast, Limonest, France) with sawdust bedding (SAFE, Augy, France) and a stainless steel wire lid. Food chow (SAFE, Augy, France) and water were provided ad libitum. The animals were maintained in colony rooms under temperature- (22°C) and humidity-controlled (55%) conditions with a 12∶12 hr light-dark cycle (lights on at 7 a.m.).

### Electrodes and Stereotaxic Surgery

Implantation surgery was conducted under Ketamine (50 mg/kg) and Xylazine (10 mg/kg) anesthesia. Mice were allowed to recover for at least a week. An 8-electrode multisite array was positioned under stereotaxic surgery. Two electrodes held in a stainless steel tube (0.4 mm dia) were positioned approximately 1.5 mm (motor cortex) and 3.0 mm (striatum) below the cranium at 0.7 mm anterior to bregma and 1.4 mm to the right of the midline suture. Three electrodes inserted in a second tube were positioned at 1.9 mm posterior to bregma and 1.4 mm right of midline, and approximately 1.2 mm (sensory cortex), 1.7 mm (CA1 of the hippocampus), and 3.5 mm (thalamus) below the cranium. These depth electrodes for local field potential (LFP) recordings were made of a single strand of 25 µm (dia) NiCr wire [11], [13]. Two additional surface electrodes (50 µm-diameter NiCr wire) for EEGs were positioned in the left frontal lobe and in the central area of the cerebellum. Finally, a 50 µm-diameter NiCr electrode was positioned in neck muscle for electromyogram (EMG) recording. Stainless steel tubes containing electrodes were used as the animal ground and reference electrode [11], [13]. Due to the choice of reference and volume conduction [14], the phenomenon of interest was observed on almost every recorded electrode, and in particular from the frontal cortex electrode.

### In vivo Electrophysiological Recording during Sleep and Data Analysis

Mice were handled and habituated to the recording chamber and room for 48 hours prior to each monthly recording. Recordings were performed for at least 4 hours during the light cycle. We were able to obtain 5 monthly recording sessions (from 2 to 6 months) from 6 R6/1 and 4 WT mice. The remaining mice were recorded only at 2 (1 R6/1 and 1 WT), 3 (3 R6/1 and 2 WT), and 4 (1 WT) different age points due to transient technical failures in the recording system. These recordings, however, allowed us to reveal the time points when a mouse displayed both the β synchrony and the clasping phenotype. Hindlimb clasping, considered as a hallmark symptom of HD, was also examined monthly on recording days.

Recordings were performed using Sciworks (Datawave Technologies, Loveland, CO). Signals were amplified (×2000), bandpass-filtered (0.1–475 Hz, Neuralynx, Bozeman, Montana) and digitized at 2 kHz. The animal’s position was tracked at 50 Hz by video camera (Camera tracker: Datawave Technologies, Loveland, CO) by means of an infrared lamp placed on the headstage. Following the experiments, thionine staining of brain sections was used to examine electrode localization. LFPs as well as EMGs were visualized for inspection using Sonic Visualiser (www.sonicvisualiser.org) to delimit the timing of various vigilance states, taking into account the mouse’s movement, EMG, wide-band mixture of 2–8 Hz (with low Hz ranges prevalent) and strong θ band. More precisely, rapid eye movement (REM) sleep was defined by low EMG, zero mouse movement, and strong theta (θactivity; slow wave sleep (SWS) by wide band (2–8 Hz) activity, zero mouse movement and low EMG; quiet wakefulness by diminished 2–8 Hz band activity, zero movement and light EMG; and active wakefulness by mouse movement recorded by camera tracker [12]. An experimenter naive to age and experimental group performed this operation. The specific segments of LFP recordings attributed to different brain states were then processed for averaged power spectra using the Matlab platform with laboratory-built programs. Fast Fourier transforms were calculated on 4-s Gaussian windows with 50% overlap. Because of the possibility that the intensity of the spectra might differ between probes and between animals, intensities at different frequencies were normalized by the sum of the overall spectra before being averaged by genotype.

## Results

### β Oscillation was Present Uniquely in R6/1 Mice

**Figure 1:**
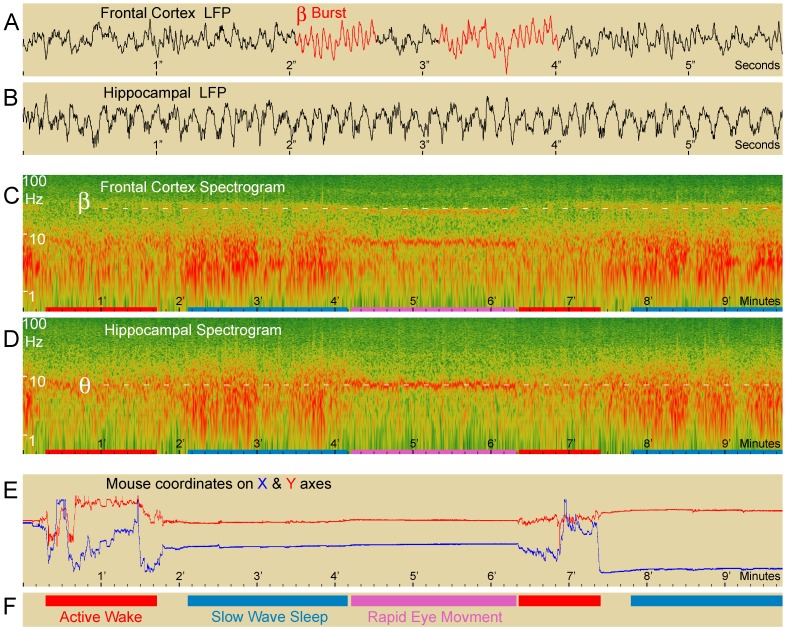
β oscillation in R6/1 Huntington’s disease transgenic mice. Typical example (9 min 30 sec of recording) of frontal cortical electroencephalogram (A) and hippocampal local field potential (B) and their respective spectrograms (C and D, log scale) in a representative symptomatic R6/1 mouse. Red and blue lines in E represent x and y coordinates of the animal’s position. With the onset of REM sleep (pink band in C, D and F), characterized by immobility and hippocampal θ rhythm (visible in D), the β band (20–35 Hz) clearly visible in the spectrogram for the frontal electrode (C), diminishes in frequency and increases in power as compared to the preceding SWS (blue band in C, D and F), which is characterized by wide 2–8 Hz bands. Different time scales were used for EEGs (A and B, REM sleep) and spectrograms (C and D) for frontal and hippocampal recordings to better illustrate the β oscillation.

**Figure 2:**
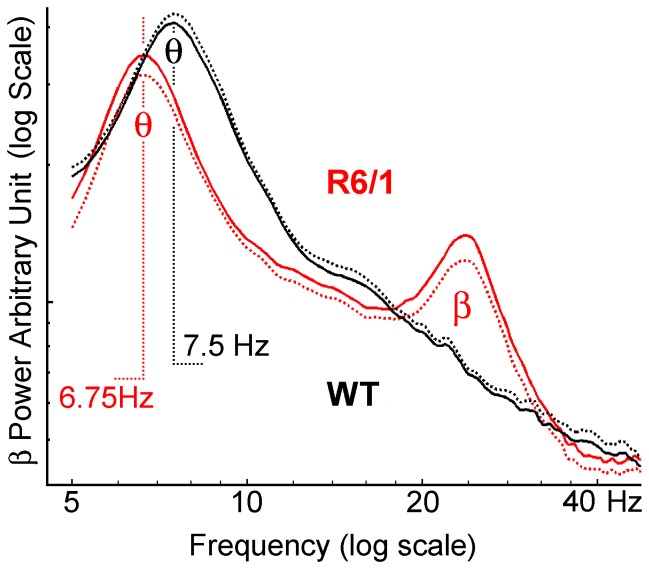
Averaged (± SEM, dotted line) spectra of REM sleep for both R6/1 (n = 10) and WT (n = 8) mice at 6 months of age. Spectra of R6/1 mice were characterized by unique 20–30 Hz β oscillation and lower peak frequency of θ oscillation (6.75 Hz instead of 7.5 Hz in WT mice).

The visual examination of raw recordings of all R6/1 mice revealed the presence of a β rhythm (∼25 Hz) in the form of clear and discrete bursts (Fig. 1A). The amplitude, duration and frequency of occurrence of these bursts varied greatly from moment to moment and contributed to “β power”. Roughly speaking, they were brief, small and rare during the active waking state, large and frequent during quiet waking and SWS, and long-lasting during REM sleep, as can be seen in Figure 1C. The β power of the raw signal was evaluated using power spectral density. Due to the choice of reference electrode and volume conduction [14] in the small mouse brain, a power peak in the β (and θ) range was visible on the spectrum plot of more or less every recorded electrode, and in particular on the frontal cortical electrode. For each of the 10 R6/1 and 8 WT mice, mean spectrum for each recording was computed during sleep and waking for all ages studied. Figure 2 represents the plot of the averages of mean spectra during REM sleep for symptomatic 6 month-old R6/1 and age-matched WT littermate mouse groups. The averaged spectra by genotype highlighted the unique presence of β activity in R6/1 mice, as well as lowered θ frequency in R6/1 as compared to WT mice.

### β Oscillation Varies with Sleep-waking States

**Figure 3:**
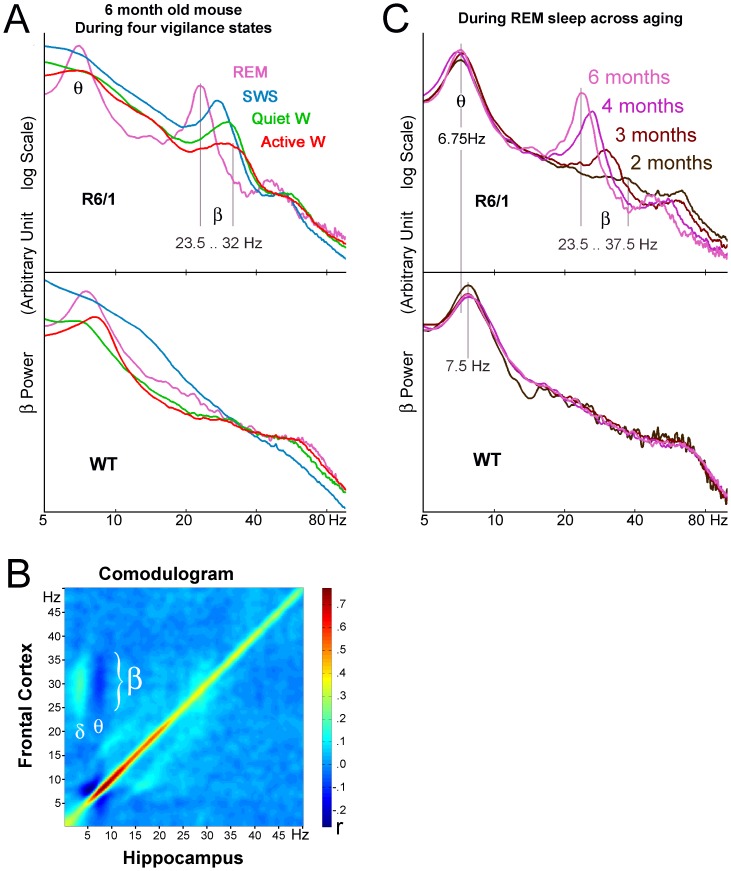
β (20–40 Hz) oscillation during different vigilance states and across aging/disease progression in R6/1 mice. Spectra from frontal electrocorticogram in a representative symptomatic 6-month-old R6/1 mouse and an age-matched wild-type littermate during four vigilance states (A): active waking (orange), quiet waking (green), slow-wave sleep (SWS, blue) and rapid eye movement (REM) sleep (pink). (B) Comodulogram between hippocampal (x-axis) and frontal cortical (y-axis) LFPs during the waking state in a R6/1 mouse. Frontal β band (20–35 Hz) activity shows a positive correlation with hippocampal δ band (2–5 Hz) activity and negative correlation with θ band (7.5 Hz) activity. (C). REM sleep spectra from frontal electrocorticogram overlaid across different ages in the same R6/1 and WT mice shown in A. The β oscillation, present only in the R6/1 mouse, shifts toward higher amplitude and lower frequency as the mouse ages. This R6/1 mouse started to exhibit clasping at 4 months of age.

Power spectral variations over time (time spectrogram) revealed a tight relationship between β power and behavioral states. Figure 1C shows 9 minutes 30 seconds of a typical recording illustrating this relationship. As can be seen in the figure, the β band appeared during SWS and intensified during REM sleep. When the animal awoke, the β component instantaneously dissipated. The inspection of a single sleep-wake transition thus sufficed to show strong β power modulation with variations in vigilance state in transgenic mice, reflecting the robustness of the phenomenon. Figure 3A shows the mean spectrum for each of the four vigilance states for representative symptomatic R6/1 and age-matched WT mice. As can be seen in the figure, the β peaks (absent in WT mice) differed not only in their surface, but also, and specifically, in their mean peak frequency. More precisely, β power increased and peak frequency decreased with diminishing vigilance, from active waking to SWS and REM sleep.

**Figure 4:**
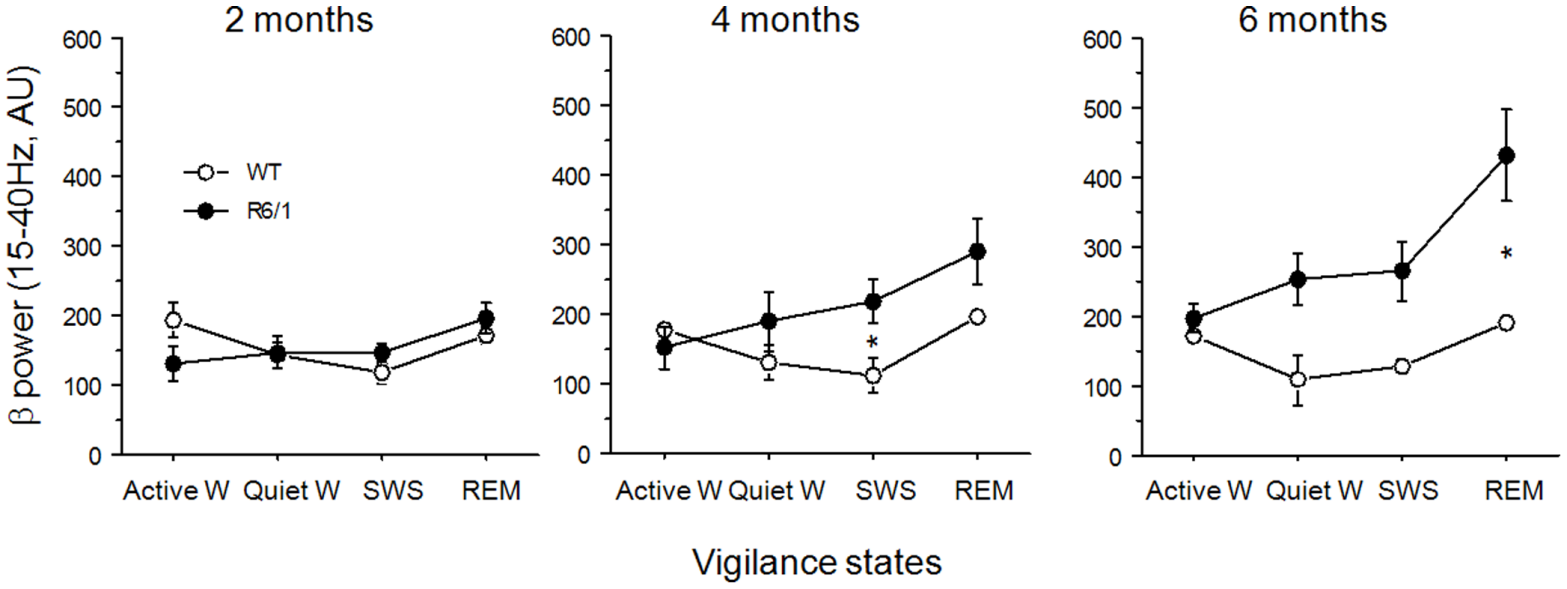
Variations of β power (15–40 Hz) across (active and quiet) waking (W), slow wave sleep (SWS) and rapid eye movement (REM) sleep at 2, 4 and 6 months. *Significant difference between genotype at p<.05. AU: Arbitrary unit.

Summed power for the β frequency range (15–40 Hz) was calculated for waking, SWS and REM sleep periods at 2, 4 and 6 months of age, and data were submitted to two-way ANOVA using genotype as a between-group factor and vigilance state as a within-group factor at each age (Figure 4). This analysis revealed that β power, irrespective of vigilance state, was significantly greater in the transgenics at 4 months (F(1,40) = 4.64, p<.05) and 6 months (F(1,41) = 16.03, p = .0003), but not at 2 months (F(1,54)<1, n.s.). The same analysis revealed vigilance state effects at 6 months (F(3,41) = 3.36, p<.05) but not at earlier ages. At 6 months of age, β power was disproportionately increased during sleep in R6/1 mice, but not during active waking state, although genotype by vigilance state interaction did not reach statistical significance (F(3,41) = 1.74, n.s.).

To verify whether this specific relationship between β power and vigilance states also exists within the active waking state, we studied the relation between β power in frontal cortical recordings and the δ and θ power of hippocampal recordings. It has been proposed that δ rhythm is associated with the resting state, while θ rhythm is associated with a high vigilance state accompanied by locomotor activity [15], [16], [17]. The waking state is composed of heterogeneous behaviors and brain states: displacement to the food and water feeders, grooming, and exploratory behaviors, punctuated by short arrests and resting periods. We thus calculated a comodulogram of these signals recorded during the waking state (Figure 3B). The comodulogram revealed that rhythmic activity in the frontal β band (20–35 Hz) was positively correlated with hippocampal δ band activity (2–5 Hz, maximal r = 0.15, p<.0001) and negatively correlated with hippocampal θ band activity (7.5 Hz, minimal r = −.10, p<.05). These results confirm that the negative relationship between vigilance level and β power; even during the wakeful state, β power was more closely associated to resting than active waking state.

### β Characteristics Evolve during Aging and Disease Progression in R6/1 Mice

As mentioned above, β synchrony was found in all of the R6/1 mice (although its age of appearance varied), but in none of the WT mice. The proportions of mice displaying β synchrony thus differed significantly between genotypes (Chi^2^ (1) = 18, p<.0001). Furthermore, and interestingly, the β peak in the same mice shifted with age toward higher amplitude and lower frequency within a given brain state (see Figure 3C, example of REM sleep spectra), while the relationship between β and vigilance state remained identical.

**Figure 5:**
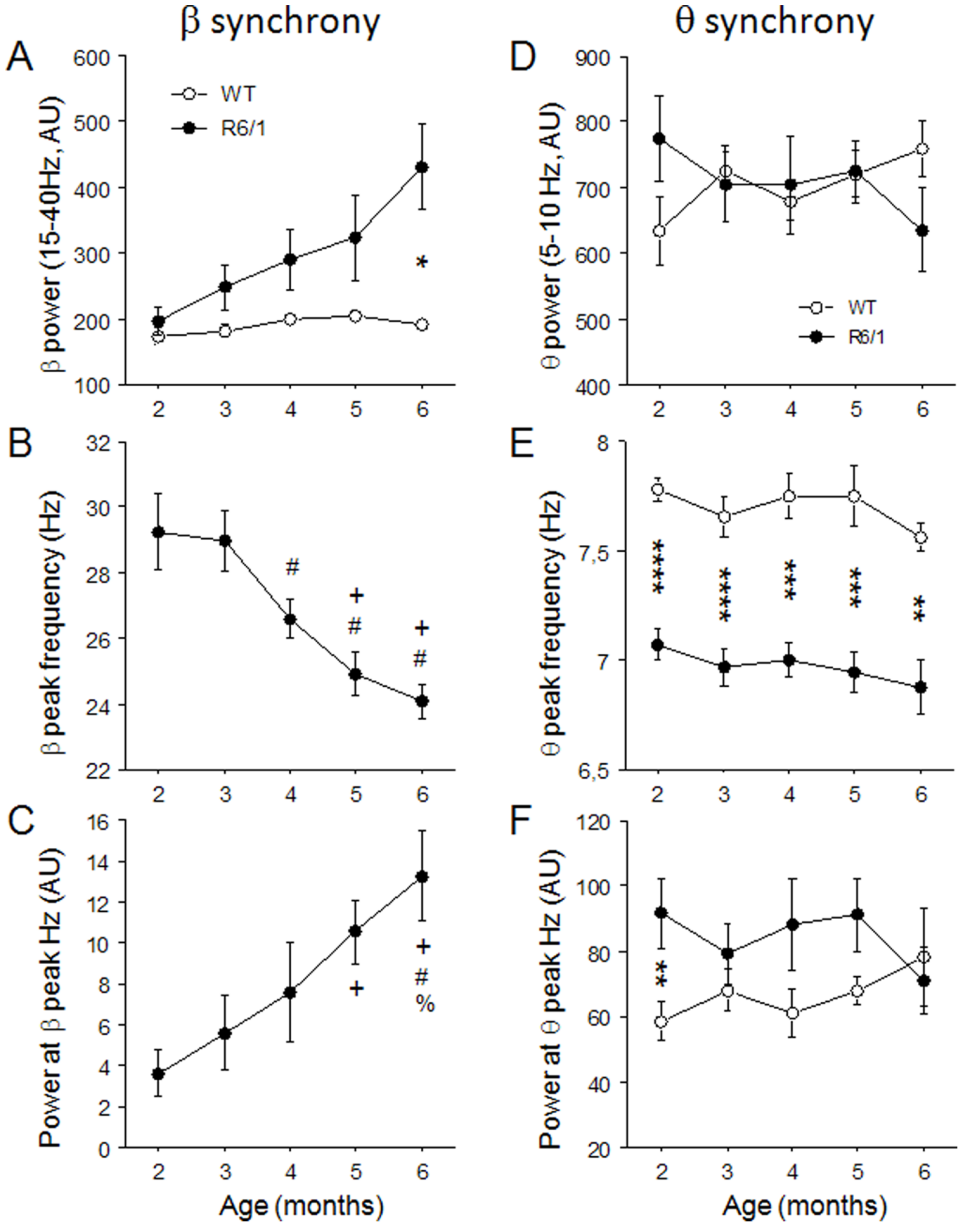
β and θ oscillations during REM sleep across age in R6/1 mice. (A,D). Summed power for β (15–40 Hz) and θ (4–8 Hz) oscillations. B and C: peak β frequency (Hz) and power at β peak are presented only for R6/1 mice because no such peak was present in WT mice; these characteristics are compared for both genotypes for θ synchrony (E,F). * Significant difference between genotypes at p<.05, ** at p<.01 and *** at p<.001. ^+^: significantly different from 2 months, ^#^: significantly difference from 3 months, ^%^: significantly different from 4 months. AU: Arbitrary unit.

In order to perform a quantitative analysis of the phenomenon, we computed summed power in the 15–40 Hz range for REM sleep, the brain state in which the β was the strongest, in each mouse. The data (summarized in Figure 5) were then analyzed using a two-way factorial ANOVA with age and genotype as main factors. This analysis revealed that β power was significantly higher in R6/1 mice across ages (F(1,47) = 66.687, p<.0001), power increased in general with age (F(4,47) = 10.382, p<.0001), mainly in R6/1 mice (age by genotype interaction, F(4,47) = 2.547, p = .051; Figure 5A). β power significantly differed between the two genotypes at 6 months of age (p<.05, Student Newman-Keuls), but not at earlier ages. As shown in Figure 5B–C, a one-way factorial ANOVA indicated that as R6/1 mice age, the β peak frequency significantly decreased (F(4.30) = 6.903, p<.001) and power at the β peak frequency significantly increased (F(4,30) = 12.78, p<.0001). Further post hoc analysis (Student-Newman-Keuls) revealed that peak frequencies at both 5 and 6 months were significantly different from that at 2 months, peak frequencies at 4–6 months different from that at 3 months. The same analysis also indicated that β power at the peak frequency at 5 and 6 months were significantly higher than that at 2 months, and that β power at 6 months differed from those at 3 and 4 months.

In contrast to the β synchrony, the characteristics (i.e. power and frequency) of 4–8 Hz activity, strongly present in the REM sleep spectra of both genotypes, remained constant across age in R6/1 mice (Figure 3C, Figure 5D–F). A two-way ANOVA with age and genotype as main factors indicated that summed power in the θ range did not differ significantly between the genotypes across all ages (Figure 5D, F(1,59)<1, n.s.). Peak θ frequency was significantly lower in R6/1 mice (Figure 5E, F(1,59) = 133.48, p<.0001), throughout all ages (age by genotype interaction, F(4,59) = 1.32, n.s.). The same analysis on power at the θ peak revealed that it was significantly higher in R6/1 mice in general (F(1,59) = 1.03, p<.05), but no age or age by genotype interaction effect was found (Figure 5F).

### β Oscillation Precedes Clasping

**Figure 6:**
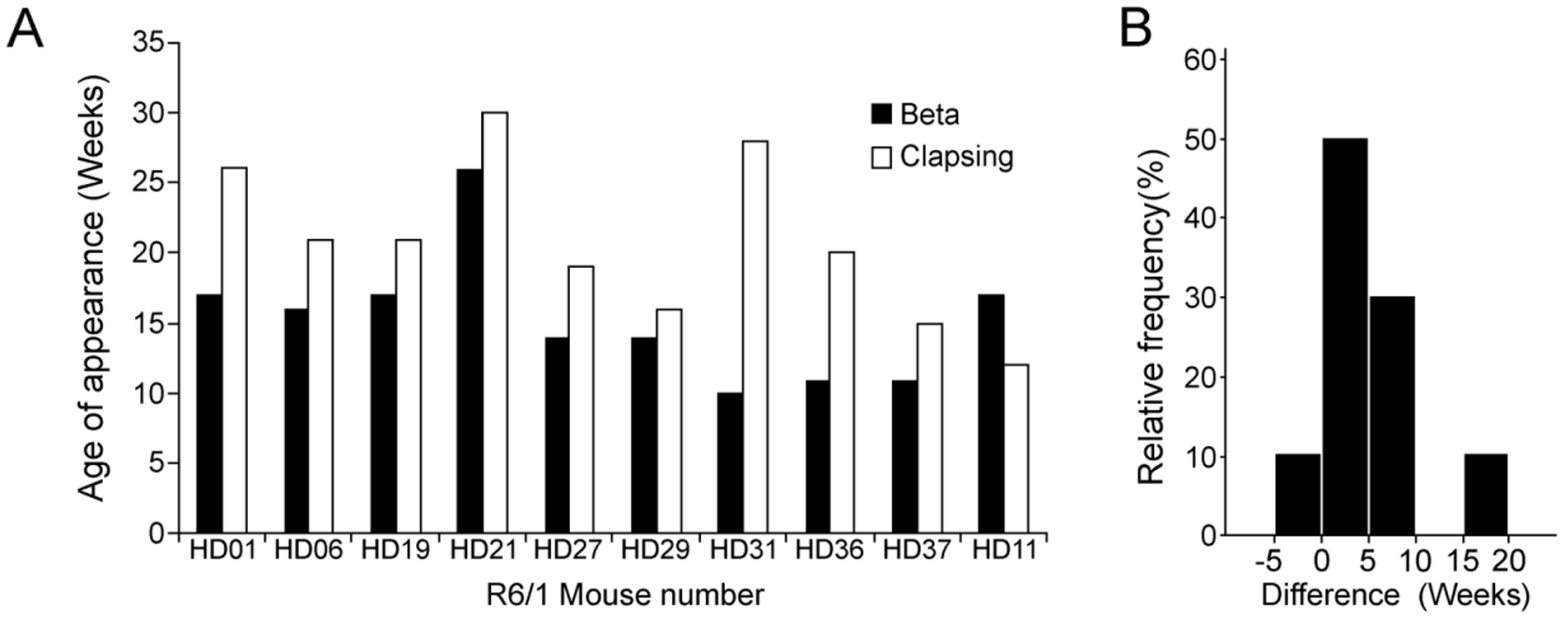
Relationship between β oscillation and hindlimb clasping in R6/1 mice. (A) Ages of β and clasping detection in ten R6/1 transgenic mice. The detection of β synchrony (present in all ten R6/1 mice) preceded clasping in 9 of them. (B) Relative frequency of precedence (in weeks) of β oscillation to clasping. Positive values signify β appearing before clasping, while negative values mean clasping appeared prior to β synchrony.

Finally, age at which β peak was detected varied in R6/1 mice, ranging from 10 to 26 weeks (mean = 16.2 weeks, SEM = 1.44, Figure 6A). Interestingly, β detection preceded neurological hindlimb clasping in nine of the ten R6/1 mice, by an average of 6.67 weeks (±1.62, Figure 6A and 6B). In the tenth R6/1 mouse, clasping preceded the β oscillation by 5 weeks (Figure 6A and 6B). This observation was confirmed by a one-way ANOVA with repeated measures, which revealed that age at β oscillation was significantly earlier than age of clasping (F(1,9) = 5.944, p = .037).

## Discussion

The goal of the present study was to look for early modification of sleep EEG in R6/1 mice, which might point to altered neuronal circuit activities pertinent to Huntington’s disease.

### Is β a Good Marker of HD Pathology in Mice?

Here, we report a progressive development of abnormal β synchrony in R6/1 mice associated with behavioral symptom development, suggesting that β may be a useful biomarker of HD pathology in this mouse model. While we were not able to finely assess the progressive development of motor symptoms in the recorded mice due to our monthly recording schedule, quantitative measures of motor coordination and strength deficits have been well documented to appear in R6/1 mice at about 20 weeks of age [18], [19], [20], and nuclear inclusions to initiate at 8 weeks [18]. The β synchrony appeared at 11–17 weeks of age, approximately the age at which cognitive deficits appear [21].

This sequence of events–mutant huntingtin inclusion; β oscillation; and cognitive and motor deficits–positions the β as one of the early events in the phenotypic alterations in R6/1 mice. These data also offer support for the idea that the β oscillation is a specific consequence of the HD mutation, and not a non-specific side effect of genetic manipulations. This point of view is reinforced by similar results obtained in R6/2 mice by Hong and colleagues [22], who described the appearance of β synchrony (termed as low γ band) while the animal was at rest. Although this study did not report the dynamic relationship of this oscillation with sleep states or its temporal evolution over the course of aging and disease progression, these oscillations in the 20–40 Hz range seem to be a result of modifications in the huntingtin protein in both R6/2 and R6/1 mice.

Strong support for this assumption comes from two recent sleep recording studies, both performed in a variant mouse line of R6/2 mice, which express increased CAG repeat length (i.e., 250 repeats). The studies on this new HD mouse line reported the appearance of γ (30–40 Hz) and high β/γ (25–60 Hz) rhythms as transgenic mice age [23], [24] (Table 1). However, contrary to our data, the β (orγ oscillation was already present at the earliest, asymptomatic age (9 weeks) in these mice, although its amplitude increased with age and symptom progression [23], [24]. These data, taken together, suggest that the pathological 20–60 Hz synchrony is common in both R6/2 and R6/1 mice, and attest to its relevance to the HD mutation.

**Table 1:** β/γ and theta synchronies during waking and sleep in Huntington’s disease transgenic mice and Parkinson’s disease (SWS: slow wave sleep).

Physiological characteristics	R6/1 mice	R6/2 mice	R6/2 mice	R6/2 mice
CAG repeats	125–135	150–165	253±1	241±5
Reference	Present study	Hong et al., 2012 [22]	Kantor et al., 2013 [23]	Fisher et al., 2013 [24]
β/γ: frequency	15–40 Hz	20–60 Hz	25–40 Hz	20–60 Hz
β/γ: waking	Absent/weak	Absent at activity Present at rest	Not reported	Strongly present
β/γ: SWS	Present	Not studied	Strongly present	Strongly present
β/γ: REM sleep	Strongly present	Not studied	Strongly present	Strongly present
β/γ: age-dependent?	Yes	Not studied	Yes	Yes
Theta frequency	Decreased	Not studied	Decreased	Decreased
Theta frequency shift with age?	No	Not studied	Yes	Yes

However, a question may still be raised as to whether the β synchrony is a consequence of distributed neuron loss or dysfunction common to different HD mouse lines, or even different neurodegenerative disorders. There are several reasons to doubt this hypothesis. First, transient β burst activity has been shown to also appear (at specific moments during procedural learning) in healthy animals free of neuron loss [25], [26]. Second, no such β oscillation has been described in other neurodegenerative diseases such as Alzheimer’s disease. Third, the characteristics of the β-to-vigilance state relationship in R6/1 mice observed here differ considerably from those seen in a rat model of Parkinson’s disease (PD) [27] as well as in PD patients [28]: in these cases, β synchrony appears during waking, but dissipates during SWS, although β increases during REM sleep as in our mice. It should be noted that β synchrony constitutes a hallmark of Parkinson’s disease [29]. This opposition seen in the relationship of β activity to waking and SWS states is sufficient to dissociate PD β from R6/1 β and to consider the pattern of β seen here to be specific to HD pathology.

Finally, the question of whether this β activity occurs in HD patients remains to be explored. To our knowledge, during sleep, two surface EEG recording studies have shown an enhancement of sleep spindle density in HD patients [9], [30], but no similar phenomenon has yet been reported. In conclusion, it seems reasonable to consider that β synchrony could be a useful biomarker with which to test the efficacy of pharmacological interventions against (sleep-related) HD pathology in both R6/1 and R6/2 mice.

### Is Hippocampal θ Frequency Shifting an Effect of HD Mutation?

Peak **θ** frequency in R6/1 mice was 0.75 Hz lower than in WT mice. However, contrary to the variation in β activity, the **θ** difference was not age-dependent. It is possible that our recording had not been performed early enough to detect the transition, and that cellular/molecular changes occurring prior to the beginning of neuronal inclusion (8–9 weeks) may be associated with a precocious **θ** shift. In addition, it is possible that non-HD-specific effects of the genetic manipulation modified the development of R6/1 mice, and produced this frequency shift independently of the HD mutation. In support of this assumption, different inbred mouse strains have shown to differ in peak **θ** frequency [31]. However, recent sleep recordings, mentioned above, have also shown a downward shift in **θ** frequencies during REM sleep in R6/2 mice–which in this case was also age-dependent [23], [24] (Table 1). Further work involving recording in R6/1 mice at earlier ages can confirm whether these findings from R6/2 mice also apply in this strain.

### The Paradoxical Relationship of β Activity with SWS and REM Sleep

The β activity seen here in R6/1 mice is absent during waking, appears with drowsiness, increases during SWS, and reaches its highest level during REM sleep. Therefore, the R6/1 β synchrony, which appears and intensifies during sleep, could simply be explained by a well-known facilitation of pathological synchronization in general by sleep [32]. Moreover, it is surprising, at first glance, that the maximum level is reached during REM sleep, which ordinarily counteracts synchronization. As an answer to this paradox, one hypothesis would be that the neuronal circuitry or mechanism on which the R6/1 β relies is strongly driven by some “sleep-promoting” mechanisms, which enter into action with SWS and intensify during REM sleep to maintain sleep. Sleep-promoting phenomena have been described, for example, as an increased threshold for awakening by sensory stimuli [33], amine depletion, and cerebral blood flow decrease in some cortical areas in humans, especially prefrontal cortex [34]. Another hypothesis would be that mesencephalic mechanisms controlling SWS, REM sleep and waking states might be disrupted by the pathology in such a way that the activation process is reversed specifically for the β circuitry. This seems unlikely because SWS-REM sleep transitions are still clearly marked in R6/1 mice despite their disorganization.

### Relation of β Activity to Vigilance States in Parkinson’s and Huntington’s Diseases

It is tempting to compare the relation of β activity to vigilance states in Parkinson’s and Huntington’s diseases. Waking and SWS states, but not REM sleep, differentiates PD β from R6/1 β. This lack of differentiation between R6/1 mice and PD in β activity during REM sleep is intriguing. The β in PD appears to require cortical activation [35], because it is strong during the desynchronized states of waking, vanishes with SWS, and re-appears with REM sleep [27]. This re-appearance is logical in PD. Nevertheless, in R6/1 mice, contrary to PD, there is no expression of β in the waking state that could re-appear during the wake-like state of REM sleep. A hypothesis to reconcile these different patterns would be that distinct elements of the cortico-basal loops, which are respectively activated and non-activated during REM sleep [34], could underlie PD and R6/1 β.

### Concluding Remarks

Our results demonstrate, first, that sleep β rhythm in a mouse model of HD may be a useful marker of pathology and sleep disturbances in this mouse model of HD, which could potentially be used to evaluate the efficacy of therapeutic approaches either in general or in correcting sleep deregulation. Second, they reveal a relation between β and sleep/vigilance states which is specific to R6/1 mice, and which is paradoxical with respect to the opposition between desynchronized REM sleep and synchronized SWS. Dissecting this unique relationship may provide a key to understanding the pathological processes in the basal ganglion in HD as well as the physiology of sleep.
